# Proteomics Mapping of the ISGylation Landscape in Innate Immunity

**DOI:** 10.3389/fimmu.2021.720765

**Published:** 2021-08-10

**Authors:** Fabien Thery, Denzel Eggermont, Francis Impens

**Affiliations:** ^1^VIB-UGent Center for Medical Biotechnology, VIB, Ghent, Belgium; ^2^Department of Biomolecular Medicine, Ghent University, Ghent, Belgium; ^3^VIB Proteomics Core, VIB, Ghent, Belgium

**Keywords:** ISG15, mass spectrometry, infection, interferon, ubiquitin-like modification

## Abstract

During infection, pathogen sensing and cytokine signaling by the host induce expression of antimicrobial proteins and specialized post-translational modifications. One such protein is ISG15, a ubiquitin-like protein (UBL) conserved among vertebrates. Similar to ubiquitin, ISG15 covalently conjugates to lysine residues in substrate proteins in a process called ISGylation. Mice deficient for ISGylation or lacking ISG15 are strongly susceptible to many viral pathogens and several intracellular bacterial pathogens. Although ISG15 was the first UBL discovered after ubiquitin, the mechanisms behind its protective activity are poorly understood. Largely, this stems from a lack of knowledge on the ISG15 substrate repertoire. To unravel the antiviral activity of ISG15, early studies used mass spectrometry-based proteomics in combination with ISG15 pulldown. Despite reporting hundreds of ISG15 substrates, these studies were unable to identify the exact sites of modification, impeding a clear understanding of the molecular consequences of protein ISGylation. More recently, a peptide-based enrichment approach revolutionized the study of ubiquitin allowing untargeted discovery of ubiquitin substrates, including knowledge of their exact modification sites. Shared molecular determinants between ISG15 and ubiquitin allowed to take advantage of this technology for proteome-wide mapping of ISG15 substrates and modification sites. In this review, we provide a comprehensive overview of mass spectrometry-based proteomics studies on protein ISGylation. We critically discuss the relevant literature, compare reported substrates and sites and make suggestions for future research.

## Introduction

### ISG15, a Ubiquitin-Like Protein of the Immune System

**Figure 1:**
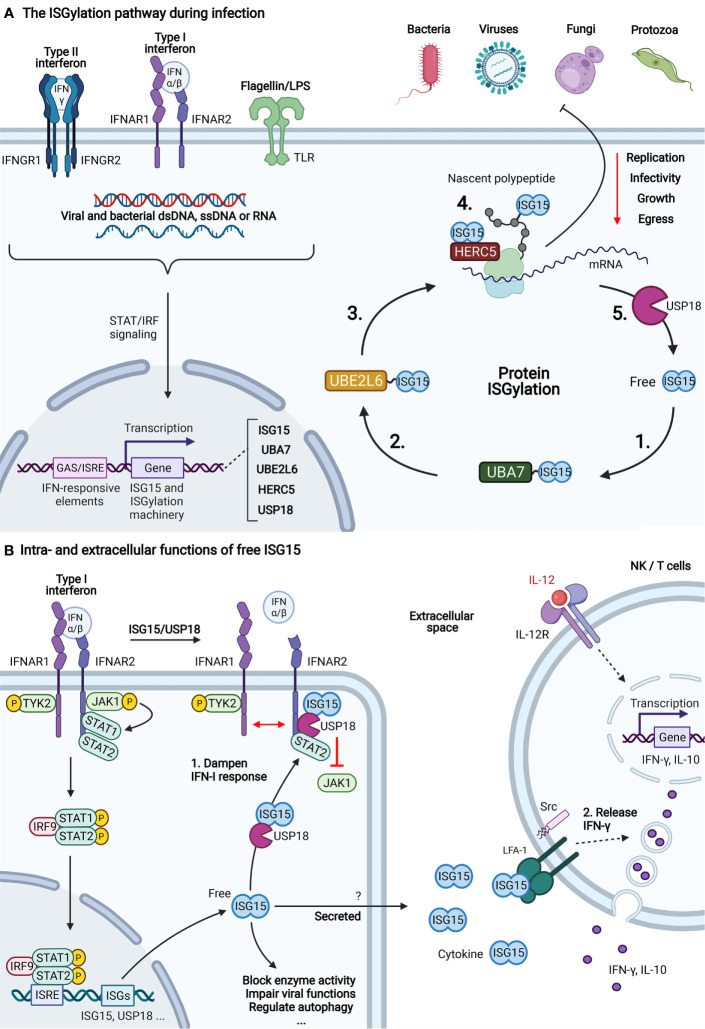
The three functions of ISG15. **(A)** The ISGylation pathway during infection. After detecting the presence of an intra- or extracellular pathogen, several antimicrobial signaling pathways lead to the expression of ISGs, including ISG15 and its conjugation machinery. The covalent attachment of ISG15 to substrate proteins, also called ISGylation, relies on the activities of an E1 (UBA7) (1), E2 (UBE2L6) (2) and E3 (hHERC5/mHERC6) (3) enzyme. The product of the ISG15-conjugation pathway is an ISGylated host or viral protein (4). Target modification by ISG15 has a widespread negative effect on the replication, growth, egress and infectivity of four major classes of pathogens, including viruses, bacteria, fungi and protozoa (1). Deconjugation is catalyzed by USP18 which releases ISG15 from its substrate (5) (6). **(B)** Intra- and extracellular functions of free ISG15. Apart from its activity as a ubiquitin-like conjugate, ISG15 also exists in a free form with functions both inside and outside the cell. (1) Type I interferon (IFN-I) signaling is the main pathway for induction of ISG15 and the ISGylation machinery, including the deISGylase USP18. Binding of IFN-I to the dimeric IFN-I receptor (IFNAR1/2) results in recruitment and activation of Janus activated kinases (JAKs) and tyrosine kinase 2 (TYK2). Consequently, the JAKs phosphorylate STAT1 and STAT2 which dimerize and form a three-protein complex with IRF9. This complex translocates to the nucleus where it binds to IFN-responsive regulatory elements (ISRE) to initiate transcription of IFN-stimulated genes (ISGs). Among these genes is *Usp18*, which, in addition to its enzymatic activity, also functions as a negative regulator of IFN-I signaling by binding to IFNAR2 (7). There, it prevents dimerization of the receptor and blocks recruitment of JAKs which puts a brake on IFN-I-signaling (8). There, it prevents dimerization of the receptor and blocks recruitment of JAKs which puts a brake on IFN-I-signaling (8). In humans, this function depends on direct interaction with ISG15 which protects USP18 from proteasomal degradation (3). Apart from its role in IFN-I signaling, free ISG15 also blocks the activity of certain enzymes, impairs viral functions by sequestering viral proteins, regulates autophagy (9). (2) When ISG15 is not conjugated to other proteins, it also becomes secreted through an unknown non-canonical secretion pathway (10, 11). In the extracellular space, it acts as a cytokine for NK and T cells where it binds to LFA-1 and induces the secretion of IFN-γ and IL-10 from secretory granules (5). The mechanism relies on a synergy between IL-12 and ISG15 where IL-12 triggers the expression of IFN-γ and ISG15 promotes the secretion of IFN-γ through downstream activation of SRC kinases. In addition, ISG15 was shown to enhance secretion of other pro-inflammatory cytokines such as CXCL1, CXCL5, IL-1 and IL-6 (12, 13). Figure created with BioRender.com.

Host cellular immunity arises from the intricate network of cell types and signaling molecules which confer resistance to pathogenic infections. As part of both the innate and adaptive immune system, interferons (IFNs) are a family of proteins released by host cells upon encounter of foreign invaders. Acting as a cytokine, IFNs signal to other cells to induce the expression of interferon-stimulated genes (ISGs) whose products control pathogenic infections. The evolutionary conserved ubiquitin‐like protein (UBL) ISG15 is one of the genes most strongly induced by IFNs and has a profound role in the antimicrobial response. For instance, ISG15 is known to counteract both viral, bacterial and fungal infections (1). To exert this function, ISG15 depends on three molecular activities which include i) negative control of interferon-α/-β signaling as a free intracellular molecule (2, 3), ii) induction of IFN-γ secretion as an extracellular cytokine and iii) ubiquitin-like protein conjugation in a process called ISGylation (4, 5) (**Figure 1**). Similar to ubiquitylation, ISGylation is mediated by the consecutive action of an E1‐activating enzyme (UBA7), an E2‐conjugating enzyme (UBE2L6) and E3 ligases (ARIH1, TRIM25 or hHERC5/mHERC6) that covalently link ISG15 to lysine residues of target proteins (14–18). In addition, ISGylation can be reversed through the action of a deconjugating protease, the ubiquitin-like carboxy-terminal hydrolase USP18 (6, 19).

Although its discovery dates back to the 1980s, ISG15 only recently regained attention as an UBL involved in a plethora of biological pathways. Aside from the immune system, ISG15 also plays a role in the progression of cancers, exosome secretion, the DNA damage response, telomere shortening, autophagy, hypoxia and ischemia (20–28). However, its main and most studied function lies within the host response against viral infection [recently reviewed in (1)]. ISG15 acts antiviral by covalently modifying host and viral proteins which interferes with viral assembly or function (29). Accordingly, mice lacking ISG15 are unable to control various pathogens including clinically relevant etiologic agents such as influenza, herpes‐, noro- and coxsackievirus (30–32). This crucial antiviral function of ISG15 is further supported by effective immune evasion strategies of specific pathogens which express ISG15 proteases (e.g. SARS/MERS virus) or interfere with ISG15 conjugation (e.g. influenza virus) (33–35). Notably, also the coronavirus pandemic led to renewed interest in ISG15 since the papain-like protease (PLpro) from SARS-CoV-2 actively targets and deconjugates ISGylated proteins to dismantle the host immune response (36, 37). In line with this, molecular inhibition of PLpro restores ISGylation levels in infected cells concomitant with reduced viral replication and virus-induced cytopathogenic effects (10).

Although ISG15 and ISGylation have been primarily characterized in the context of viral infection, it was recently shown that ISG15 also protects against infections by intracellular bacteria such as *Listeria monocytogenes* (*L. monocytogenes*) and *Mycobacterium tuberculosis* (*M. tuberculosis*) or predominantly extracellular bacteria like *Pseudomonas aeruginosa* (38–40). Concordantly, individuals with an inherited ISG15 deficiency show an increased susceptibility to weakly virulent *M. tuberculosis*, a condition known as Mendelian susceptibility to mycobacterial disease (MSMD) (4). Although this phenotype was first ascribed to the extracellular function of ISG15, it was later shown that ISG15 conjugation is also upregulated during *M. tuberculosis* infection *in vivo* (39). Likewise, our laboratory and others have shown that increased levels of ISGylation protect against *L. monocytogenes in vitro* and *in vivo* (38). Meanwhile beyond bacteria, ISGylation was also found to be critical to control *Toxoplasma gondii* (*T. gondii*) infection in human cells (41).

Clearly, ISG15 and ISGylation harbor a broad antimicrobial effect against several major classes of pathogens. Evidently, this raises the question how one single protein modification can target such a diverse set of disease agents. One model suggests that cotranslational modification of newly translated proteins is the basis of ISG15’s far-reaching antimicrobial function. Indeed, the dominant ISG15 E3 ligase HERC5 associates with polyribosomes and preferentially modifies proteins that are being synthesized during the active stages of the conjugation machinery (29). Considering that during viral infection mainly viral proteins are translated, ISGylation will preferentially target viral proteins thus disturbing their folding and activity. As such mechanism does not require affinity for specific viral components, ISG15 cotranslational tagging likely contributes to the ability of ISG15 to counteract a broad range of viruses. While this model contributes to our understanding of the antiviral activity of ISG15, it does not explain how ISGylation counteracts bacterial infections. In addition, viral infection or type I interferons (IFN-I) signaling also leads to strong ISGylation of host proteins for which the functional role is still unknown. To gain insights into the biological implications of ISGylation, research has focused on identifying the targets and sites of ISGylation induced upon infection.

Like many other protein post-translational modifications (PTMs), research on UBLs has benefited greatly from recent advances in mass spectrometry (MS)-based proteomics. Aside from shotgun proteomics experiments to profile protein changes upon ISG15 modulation (42–44), several MS screens were performed in the past two decades to identify targets and sites of ISG15 with increasing levels of ingenuity, but also with their own set of shortcomings. In this review, we provide a comprehensive overview of MS-based studies on protein ISGylation. We critically discuss the relevant literature and make suggestions for future developments and research.

### MS-Based Approaches to Identify ISG15 Substrates

Despite its early classification as an UBL, it was not until 2002 when the first substrates of ISG15-conjugation were discovered. Following immune challenge of murine macrophages with bacterial DNA, Hamerman and colleagues identified Serpin2a as a bacteria-induced host protein with unexpected higher molecular mass forms on western blot (45). The authors expressed a tagged version of Serpin2a in macrophages followed by immunoprecipitation (IP) and separation on SDS-PAGE to isolate these modified forms of Serpin2a. Subsequent MS analysis then revealed mouse ISG15 as the PTM that conjugates to Serpin2a during macrophage activation. One year later, four additional ISG15-substrates were reported using a high-throughput western blot screen on human thymus samples (46).

In the years that followed, different research groups continued using untargeted MS-based approaches to identify targets of ISGylation. In 2005, a research team led by Robert M Krug was the first to report a comprehensive catalog of 156 human ISGylated proteins (47). The authors overexpressed doubly tagged His-FLAG-ISG15 along with E1/E2 enzymes in HeLa cells treated with IFN-I. ISGylated proteins were isolated from cellular lysates by affinity pulldown and separated by SDS-PAGE prior to their MS identification from specific gel bands. *Bona fide* ISGylated proteins were distinguished from potential contaminants by applying the same procedure on untransfected control cells. Similarly, Takeuchi and colleagues relied on overexpression of FLAG-ISG15 and the ISGylation machinery (E1/E2 enzymes) in HeLa cells to identify six additional substrates of ISG15 (48).

Although new insights were gained into the functions of ISG15, both studies suffered from the use of transient transfection to overexpress the ISGylation machinery. This was known to introduce artefacts through modification of collateral proteins that are not endogenous ISG15-conjugates (29). To overcome these limitations, alternative strategies were devised based on antibodies recognizing endogenous ISG15 or on cell lines stably expressing tagged ISG15. Wong et al. engineered A549 cells to express FLAG-ISG15 which was leveraged to isolate ISGylated proteins from IFN-I-treated cells (49). They identified 168 ISGylation targets of which 24 were biochemically validated. Moreover, the use of A549 instead of HeLa cells elegantly expanded the repertoire of ISG15 targets in epithelial cells. Also during that time, the first ISG15 substrates derived from professional immune cells were uncovered thanks to the lab of Dong-Er Zhang. Here, the authors relied on antibodies raised against human and mouse ISG15 to isolate and map ISG15 substrates in human U937 monocytes and *Usp18^-/-^* mouse embryonic fibroblast (MEF) cells treated with IFN-I (50). In total, 76 ISGylation targets were discovered of which 21 were found in both human and mouse cells, suggesting a core set of ISGylated proteins shared across different species and cell types. More recently, Care et al. applied the same antibody-based strategy to draft the first catalog of ISG15 substrates in primary human cells (51). The authors investigated how ISG15-conjugation is involved in the maturation of plasmablasts into immunoglobulin-secreting plasma cells. To this end, primary B cells were isolated from human donors, treated with IFN-I and immunoprecipitated with anti-ISG15 antibodies. MS analysis and comparison with control-IP samples revealed 52 ISGylated substrates, several of which are known regulators of B cell maturation.

Nonetheless, the identification of endogenous ISG15 substrates through IP remains suboptimal due to the inefficiency of ISG15 antibodies to enrich for ISGylated proteins (47, 52). As a result, Yan et al. recently developed a novel approach to map ISG15 targets based on adenoviral delivery and stable overexpression of FLAG-ISG15 into primary *Isg15^-/-^* adipocytes treated with LPS (52). In this way, potent anti-FLAG antibodies could be used to fish for ISGylated proteins, similar to the early screens of Takeuchi and Wong. Following standard IP and MS analysis, the authors eventually identified 527 murine ISG15 substrates, hitherto the highest number of ISGylated proteins reported in a single experiment. One of the reasons for their high coverage is the incorporation of state-of-the-art proteomics tools, including peptide isotopic labeling by Tandem Mass Tags (TMT) and high-resolution orbitrap MS analysis.

Together, these studies made important contributions to the field by expanding the landscape of potential ISGylation functions beyond the scope of microbial infections. Nevertheless, ISG15’s antimicrobial activity is still considered as its foremost function which prompted additional proteomics studies in the context of infection. In 2015, Radoshevich et al. demonstrated that ISG15 counteracts infection with the intracellular bacterial pathogen *L. monocytogenes in vitro* and *in vivo* (38). To work out the molecular determinants underlying the antilisterial activity, the authors developed a quantitative proteomics approach to identify ISG15 substrates. Using HeLa cells stably expressing FLAG-His-ISG15, they isolated ISGylated proteins by His pulldown and quantitatively measured the levels of ISG15-conjugates with Stable Isotope Labeling by Amino acids in Cell culture (SILAC). Unlike previous screens where in-gel digestion was used without any isotopic labeling approach, this report was the first to combine contemporary in-solution sample preparation with SILAC-based quantification. With their approach, the authors identified 42 new ISG15 substrates of which two were validated at the endogenous level during *L. monocytogenes* infection. Similarly, Bhushan and colleagues investigated the role of ISGylation signaling during autophagy-mediated growth restriction of intracellular *T. gondii* (41). The authors found that ISG15 is part of the proxisome of the Autophagy Gene 5 (ATG5) where it mediates recruitment of autophagy adaptors to the pathogen-containing vacuole (PV). Accordingly, deletion of ISG15 blocked PV-recruitment which impaired IFN-γ-dependent control of *T. gondii*. To discern the function of ISG15-conjugation during this process, the authors mapped ISG15 substrates in A549 cells upon IFN-γ treatment. Their protocol used A549 cells stably expressing wild-type or non-conjugatable ISG15 (control) that were subjected to ISG15 IP and on-bead trypsin digestion followed by MS analysis. 239 ISGylated proteins were identified, making up the first list of ISGylated proteins induced upon IFN-γ treatment in an epithelial cell line. Finally, one study identified ISG15 substrates during influenza A viral infection (53). Here, A549 cells were cultured in presence or absence (control) of the virus to induce a physiological ISGylation response. After 24h, cells were lysed and ISGylated proteins were pulled down prior to on-bead trypsin digestion and LC-MS/MS analysis. Among 22 ISGylated proteins, the authors picked up the influenza A non-structural protein 1 as a known viral target of ISGylation (54, 55).

Together, just 20 years after the initial discovery of the first ISG15 substrate, MS-based methods led to the successful identification of hundreds of ISGylation substrates in various cell types upon different stimuli. However, many of these studies relied on artificial systems that used ectopic overexpression of ISG15 in cultured cells, leading to overexpression artefacts, and on treatment with IFNs which could induce a stronger ISGylation response than what is physiologically induced during infection. In addition, none of the above screens was able to pinpoint the exact modification site on substrate proteins, making it difficult to investigate how ISG15-conjugation affects the function of the identified substrates.

### Proteome-Wide Mapping of ISG15 Modification Sites

To overcome the inability to map ISG15 modification sites and inspired by recent developments in the ubiquitin field, we recently devised a novel approach to study protein ISGylation (56). When trypsin cleaves proteins into peptides, also conjugated ISG15 becomes proteolyzed which leaves a diglycine tag (GG) attached to the original modified lysine residue. As a result, peptides that were modified by ISG15 can be isolated from the bulk of unmodified peptides using anti-K-ϵ-GG antibodies. Hence, the modified peptides including the exact site of modification can be identified by LC-MS/MS. Without any further comparison, however, GG peptide pulldown is unable to distinguish ISG15 sites from ubiquitin and NEDD8 sites since trypsin digestion leaves the same diglycine adduct for all three PTMs. One evident solution is to include an *Isg15^-/-^* control condition where all enriched sites either correspond to NEDD8 or ubiquitin sites. In this way, sites that are uniquely identified in the wild-type correspond to *bona fide* ISG15 sites. Using this comparative approach, we were able to map 930 ISG15 sites on 434 proteins in the liver of mice infected with *L. monocytogenes*, the first study reporting on the proteome-wide identification of ISG15 modification sites, directly in an *in vivo* model of bacterial infection.

Last year, the group of Benedikt Kessler published a similar strategy to map ISG15 modification sites in the chronic myeloid leukemia (CML)-derived cell line HAP1 (57). Here, the authors used *Usp18* ^-/-^ cells instead of *Isg15* ^-/-^ cells to control for the ubiquitin/NEDD8 signal spillover. Knockout of USP18 as the major deISGylation enzyme leads to massive accumulation of ISGylated proteins after IFN-I treatment. Hence, sites differentially regulated between *Usp18* ^-/-^ and wild-type cells correspond to actual ISG15 sites, assuming that ubiquitylation and NEDDylation are not affected in *Usp18* ^-/-^ cells. Their approach eventually uncovered 796 ISG15 modification sites on 476 substrate proteins. In addition, the authors validated 110 of the identified targets in the same cellular system by ISG15 IP followed by LC-MS/MS. Together, both approaches led to the joint discovery of 679 USP18-dependent substrates of ISG15.

Finally, a recent proteomics study on ISGylation generated the first list of porcine ISG15 substrates in a porcine alveolar macrophage cell line treated with IFN-I (58). Similar to previous studies, the authors relied on an antibody against porcine ISG15 to isolate ISGylated proteins prior to in-solution digestion and MS analysis. Even without specific enrichment, MS analysis allowed to identify GG-modified peptides and map 190 ISGylation sites on 97 substrate proteins. Evidently, this study demonstrates the capacity of contemporary MS-based proteomics to map ISG15 modification sites directly after ISG15 pulldown, albeit with a lower efficiency compared to standard GG-peptidomics.

## Common Substrates and Future Perspectives

**Figure 2:**
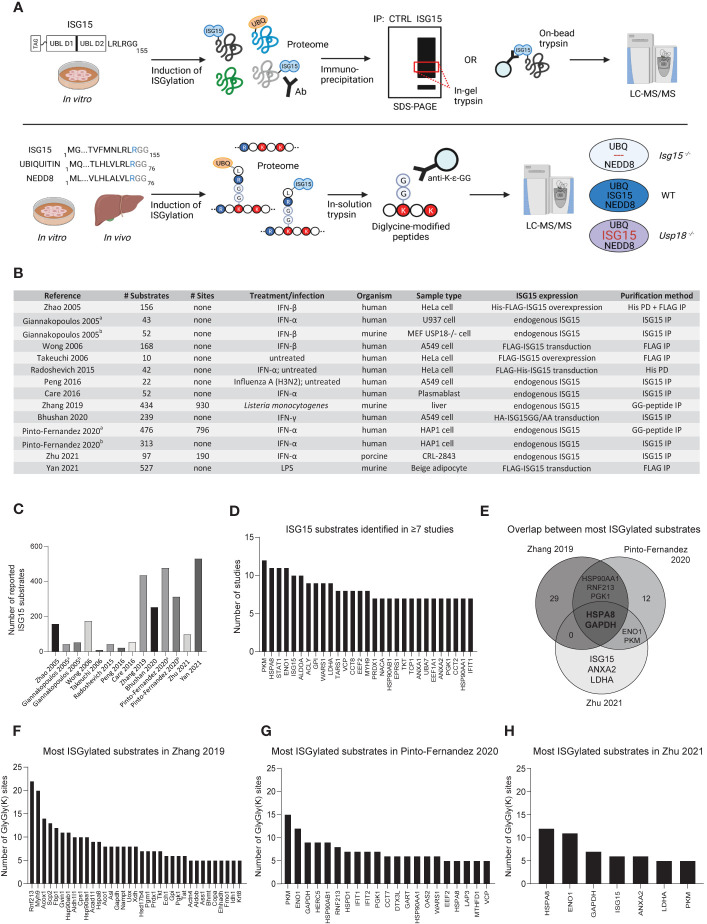
MS-based discovery of ISG15 substrates. **(A)** Commonly used workflows to identify ISG15 substrates by mass spectrometry. Upper panel: many studies rely on pulldown of ISGylated proteins from induced cells using antibodies or resins against endogenous or overexpressed (tagged) ISG15. Pulled down proteins are separated by SDS-PAGE to trypsinize specific gel bands. Alternatively, proteins are digested directly on-bead. In both cases, the released peptides are analyzed by liquid chromatography-tandem mass spectrometry (LC-MS/MS). Non-specific binders can be controlled by parallel analysis of cells expressing no ISG15 or non-conjugatable ISG15 (e.g. ISG15AA) or by using an antibody isotype control. Lower panel: to identify exact sites of ISG15 modification, recent studies applied diglycine (GG) peptide enrichment from induced cells or tissues. Here, trypsin digestion of extracted proteins generates GG-modified peptides derived from conjugated ISG15, ubiquitin or NEDD8. These GG-modified peptides can be captured with specific anti-K-ϵ-GG antibodies prior to their identification by LC-MS/MS. To distinguish ISG15 modification sites from ubiquitin and NEDD8 sites, genetic controls are included such as *Isg15^-/-^* mice or *Usp18^-/-^* cells. Consequently, comparison with a wild-type condition allows to pinpoint *bona fide* ISG15 sites based on their absence in *Isg15^-/-^* or increased abundance in *Usp18^-/-^* conditions. Figure created with BioRender.com. **(B)** Overview of MS-based proteomics studies reporting ISG15 substrates, listing relevant information on the number of identified substrates and the experimental design. **(C)** Bar chart showing the increase of identified ISG15 substrates in the different studies over time. **(D)** Bar chart showing 29 ISG15 substrates that were reported in ≥7 studies. **(E)** Venn diagram showing the overlap between the most modified ISG15 substrates listed in panel **(F–H)**. **(F–H)** Bar charts showing the most modified ISG15 substrates (substrates with ≥5 sites) in the three GG-peptidomics screens.

In the above studies two strategies were used to map ISG15 substrates: i) enrichment of ISGylated proteins on the protein level and ii) pulldown of GG-modified peptides to identify the protein along with the modification site(s) (**Figure 2A**). Together, both strategies led to the discovery of 1,563 ISG15 substrates of which 64 were validated by orthogonal approaches (**Supplementary Table S1**), with a coverage increasing over time thanks to more performant MS technologies and improved methods to study ISG15 (**Figures 2B, C**). For example, GG-peptidomics alone resulted in more identified ISGylation substrates than all previous approaches combined. By looking at the overlap across all screens, 29 ISG15 substrates were identified in half of the studies, suggesting that despite the different species, cell types and strategies employed, there is a degree of selectivity for certain ISG15 substrates (**Figure 2D**). Among these 29 proteins are many metabolic proteins, especially glycolytic enzymes, and ISGs such as STAT1 and IFIT1, in line with previous reports (44, 52, 56, 59).

Aside from protein substrates, three studies additionally uncovered 1,916 ISG15 modification sites (**Supplementary Table S2**). However, unlike SUMOylation (60), these studies did not report any sequence motif that would drive the specificity for ISG15 conjugation (56, 57). We ranked ISG15 substrates based on their number of identified GG sites, which allowed us to highlight the top most modified ISG15 substrates in each study (**Figures 2E–H**). By looking at the overlap, the molecular chaperone HSPA8 and the glycolytic enzyme GAPDH were found as most modified ISG15 substrates. GAPDH was found in six out of fourteen screens, while HSPA8 was found in eleven screens, so far without any further characterization. Likely, ISGylation of these key proteins regulates their activity or interaction state, influencing the cellular processes in which they are involved.

Despite the gain of knowledge through ISG15 site-seeing, studies by Zhang and Pinto-Fernandez relied on genetic approaches with *Isg15^-/-^* animals or *Usp18^-/-^* cells to discern *bona fide* ISG15 sites. Since both proteins are regulators of IFN-I signaling, but also other processes (3, 61) (**Figure 1**), their absence could potentially introduce artefacts which might also affect the identification of ISG15 sites. Future studies will need to address this caveat by developing novel genetic approaches that preserve both IFN-I signaling and endogenous ISG15 conjugation. In analogy to strategies employed for the identification of SUMO sites (62, 63), one could envision to mutate ISG15’s C-terminal sequence to LRL**K**GG, which after endopeptidase Lys-C digestion will generate ISG15-derived GG-modified peptides that can be specifically enriched and identified by MS. However, such genetic approaches cannot easily be used in a clinical setting.

To overcome the need for genetic approaches and expand the repertoire of applicable samples, alternative approaches could be based on novel antibodies or technologies that combine enzymatic and chemical methods. Unlike trypsin digestion which generates the same GG-modified peptides for ubiquitin, NEDD8 and ISG15, digestion with endopeptidase Lys-C leads to remnants that are specific for each UBL. Hence, antibodies developed against this fragment could be used to fish for ISG15-modified peptides. In the case of ubiquitin, this strategy led to the proteome-wide identification of 63,000 unique modification sites (64). Another elegant strategy would be to include a treatment with USP2 (a ubiquitin-specific protease) ahead of trypsin digestion and GG-peptide enrichment to deplete the sample from ubiquitin-modification sites. Similarly, the specificity of proteases such as USP18 could be leveraged to reveal free lysine residues at ISG15 modification sites available for chemical labeling and enrichment by affinity precipitation (65) or diagonal chromatography (66). Beyond label-free quantification, improvements in quantification methods also have the potential to increase site identification by GG-peptidomics, as demonstrated by the incorporation of SILAC and TMT-labeling in several ubiquitin studies (67, 68).

## Author Contributions

FT and DE wrote the manuscript. DE conceived **Figure 1**. FT conceived **Figure 2** and the supplementary tables. FI supervised FT and DE and edited the manuscript. All authors contributed to the article and approved the submitted version.

## Funding

FI acknowledges funding from Odysseus grant G0F8616N from the Research Foundation Flanders (FWO) and ERANET Infect-ERA BacVIRISG15. DE is supported by the FWO PhD fellowship 1167221N for fundamental research.

## Conflict of Interest

The authors declare that the research was conducted in the absence of any commercial or financial relationships that could be construed as a potential conflict of interest.

## Publisher’s Note

All claims expressed in this article are solely those of the authors and do not necessarily represent those of their affiliated organizations, or those of the publisher, the editors and the reviewers. Any product that may be evaluated in this article, or claim that may be made by its manufacturer, is not guaranteed or endorsed by the publisher.
